# The best of both worlds: deep brain stimulation or high-frequency focused ultrasound for tremor refractory syndromes

**DOI:** 10.1055/s-0045-1808084

**Published:** 2025-07-01

**Authors:** Carina França, Rubens Gisbert Cury

**Affiliations:** 1 Universidade de São Paulo, Faculdade de Medicina, Departamento de Neurologia, São Paulo SP, Brazil.; 2 Hospital Israelita Albert Einstein, Departamento de Neurologia, São Paulo SP, Brazil.

## INTRODUCTION

In this issue of Arquivos de Neuropsiquiatria, there was a much-needed debate between two advanced techniques for the treatment of refractory tremor, mainly Parkinson's disease (PD) and essential tremor (ET). At first glance, both deep brain stimulation (DBS) and high-frequency focused ultrasound (HIFU) seem to be direct competitors. However, looking more closely, one can see they are, in fact, complementary procedures, since they offer a more comprehensive solution than either would alone.

## Body of scientific evidence available

**Figure 1:**
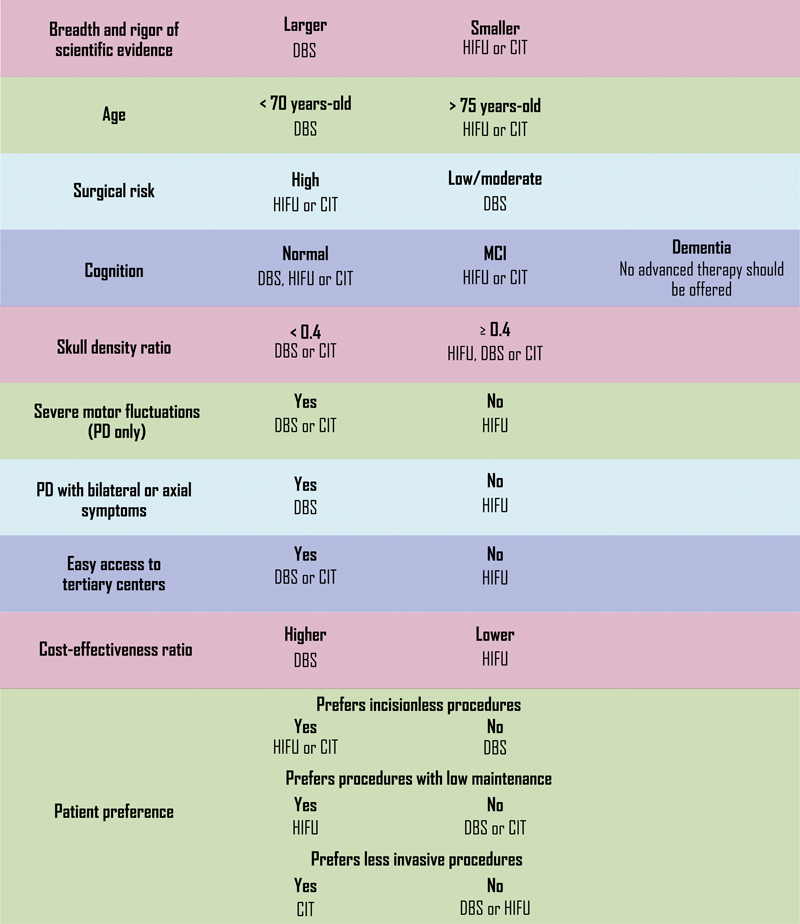
Abbreviations: CIT, continuous infusion therapy; DBS, deep brain stimulation; HIFU, high-frequency focused ultrasound; MCI, mild cognitive impairment. Medically-refractory tremor decision points for advanced therapies.

To this day, DBS is undoubtedly the advanced technique with the strongest scientific evidence of safety and efficacy for both ET and PD 1 2 ( Figure 1 ). There is an abundance of studies proving it is safe and effective, even when observing long term outcomes larger than 10 years. 2 3 Additionally, there is the great advantage of being a treatment that can be done bilaterally and simultaneously. 3 Although bilateral HIFU has been shown to be safe and feasible in recent trials, 4 it cannot be performed on both sides on the same day and it is still approached with caution, 5 6 7 particularly in light of past bilateral ablative procedures leading to frequent and irreversible side effects. 8

Furthermore, DBS allows for individualized programming adjustments over time, enabling clinicians to optimize symptom control as the disease progresses. In contrast, HIFU's effects are static, which may limit adaptability in cases of symptom fluctuations.

However, we must bear in mind that DBS is a much older technique, and it has been improved with time; in the same fashion, results with HIFU also tend to improve. 9 Improvements in target selection (e.g., diffusion-weighted imaging tractography) and refinements in staged bilateral procedures may further enhance its safety and efficacy.

## Patient's intrinsic characteristics: age, surgical risk, cognition and skull density ratio

The surgical complexity of DBS is unquestionably greater than that of HIFU: the medical training required to achieve good results is lengthier, the procedure is longer, with longer hospitalization required, and the risk of infection and bleeding should not be dismissed. 10 Therefore, patients with lower preoperative cognitive ability and more severe clinical comorbidities could be better suited with HIFU ( Figure 1 ). Regarding age, although a number of studies suggest no increased risk of complications in older patients, 11 it is customary to favor HIFU when advising patients over 75-years-old ( Figure 1 ). Its main intrinsic limitation is skull density ratio, that should be ≥ 0.4 and might prevent some patients from being eligible 12 ( Figure 1 ).

## Geographic and sociodemographic concerns

Considering device and surgical costs and the need for constant programming visits, DBS cannot be considered an easy access therapy. Even when overlooking the costs, if a patient cannot maintain a proper follow up after surgery, they will not have the expected improvement in quality of life. Therefore, if a patient resides far from the DBS center, particularly considering individuals with mobility issues, then other approaches should be prioritized ( Figure 1 ).

The cost-effectiveness of HIFU seems to be better than DBS, which could be a tilting point for underdeveloped healthcare systems, although access remains a challenge in certain regions due to magnetic resonance imaging (MRI) availability and specialized training requirements ( Figure 1 ). 12

## Patient preferences

In an era of widespread medical knowledge, patients tend to form their opinions before proper medical guidance, and “self-indicate” the kind of treatment they consider more suitable for their needs. While respecting patients' wishes and personal views, it is the clinician's role to make a stance regarding what treatment should best fit a particular patient, considering scientific evidence.

It is common for patients to dread invasive procedures, and the initial notion that HIFU is noninvasive might have helped to increase its appeal. However, it is important to highlight this is an incisionless procedure, but not a noninvasive one, since there is irreversible brain lesion. 7 On the other hand, continuous infusion therapiy (CIT) could be considered a less invasive option, as although it involves a breach of skin barrier, it does not require direct intervention in the central nervous system ( Figure 1 ).

Another point of division in favor of HIFU is the need for programming sessions after DBS. Is it unquestionably easier to undergo a procedure and leave the hospital with the best possible benefit, rather than enduring multiple programming sessions to achieve improvement ( Figure 1 ). Nonetheless, it is precisely the malleability of DBS that makes it unlikely that its results could be matched by a simple lesion.

In conclusion, when managing a heterogeneous group of patients, it is essential to carefully consider each patients' individual characteristics in order to provide the most personalized and effective advice. The availability of both DBS and HIFU depends on several factors. We also need to keep in mind that these treatments could be used on the same patient, depending on their demand at the time. Therefore, it is crucial for any physician treating tremor patients to be well-versed in advanced techniques, ensuring that they receive the best possible care.
